# Measuring functioning and disability using household surveys: metric properties of the brief version of the WHO and World Bank model disability survey

**DOI:** 10.1186/s13690-021-00654-9

**Published:** 2021-07-12

**Authors:** Carla Sabariego, Carolina Fellinghauer, Lindsay Lee, Aleksandra Posarac, Jerome Bickenbach, Nenad Kostanjsek, Somnath Chatterji, Kaloyan Kamenov, Alarcos Cieza

**Affiliations:** 1 Department of Health Sciences and Medicine, University of Lucerne, Lucerne, Switzerland; 2 Swiss Paraplegic Research, Nottwil, Switzerland; 3 Center for Rehabilitation in Global Health Systems, WHO Collaborating Center, University of Lucerne, Lucerne, Switzerland; 4 Sensory Functions, Disability and Rehabilitation, Department of Noncommunicable Diseases, World Health Organization, Avenue Appia 20, 1211 Geneva, Switzerland; 5 Africa East Human Development, The World Bank, Washington, USA; 6 Department of Information, Evidence and Research, World Health Organization, Geneva, Switzerland; 7 Department of Data and Analytics, World Health Organization, Geneva, Switzerland

**Keywords:** Model disability survey, Disability, Functioning, Rasch model

## Abstract

**Background:**

The Model Disability Survey (MDS) is the current standard recommended by WHO to collect functioning and disability data. Answering calls from countries requesting a version to be implemented as a module that could be integrated into existing surveys and be used for monitoring disability trends and for data disaggregation, WHO developed the brief MDS. The objectives of this paper are to evaluate the metric properties of the disability metrics generated with the Brief MDS and the precision of the Brief MDS in comparison with the full MDS.

**Results:**

The partial credit model, a unidimensional model for polytomous data from the Rasch family, was applied to evaluate psychometric properties using data from national MDS implementations in Chile (*N* = 12,265) and in Sri Lanka (*N* = 3000). The Brief MDS generates valid metrics for measuring disability, from the perspectives of capacity and performance, thereby achieving good levels of measurement precision in comparison with its full counterpart.

**Conclusion:**

Given the scarcity of valid functioning and disability modules for household surveys, the Brief MDS represents a milestone in disability measurement. The Brief MDS is currently used by countries to monitor disability trends over time, which is especially important to evaluate the impact of health policies and public health interventions, to disaggregate indicators of the Sustainable Development Goals, and to monitor the implementation of the UN Convention on the Rights of Persons with Disabilities (CRPD).

**Supplementary Information:**

The online version contains supplementary material available at 10.1186/s13690-021-00654-9.

## Background

In 2011, the World Health Organization (WHO) and the World Bank initiated the development of the Model Disability Survey (MDS), answering calls for sound tools for measuring functioning and disability at the population level. The MDS [1] is fully grounded in the WHO International Classification of Functioning, Disability and Health (ICF) [2] and reflects three core WHO principles [3]. First, that disability is a universal human experience; secondly, that disability is the outcome of an interaction between these and contextual factors and not simply a consequence of a health condition or impairment; and thirdly that disability is a matter of degree, ranging from low to very high levels of severity. Responding to these principles, the MDS relies on representative samples of the general population, compares mild, moderate, and severe levels of disability across a range of health conditions, and fully recognizes the impact of the physical, human-built, attitudinal and socio-political environment on disability (i.e. contextual factors).

The major goal of implementing a dedicated survey like the MDS is to generate rich data to improve the lives of persons with health conditions who experience disability to different degrees. The need for the kind of data MDS can generate is recognized in the literature. For example, Richards et al. have stressed the importance of going beyond mortality data to capture functioning and disability in order to understand the true impact of non-communicable diseases (NCDs) on people’s lives [4]. This evidence is also important to inform policies that are relevant to specific populations, such as persons with mental disorders [5, 6] or those experiencing disability of different degrees of severity [7, 8]. MDS has already been used to identify gaps in disability modules of health surveys [9], to guide the standardisation of national data collection efforts in Germany [10] and to enrich disease-specific surveys, as is the case of the International Spinal Cord Injury survey [11]. MDS data has also been used to analytically demonstrate the value of having representative samples of the general population when collecting functioning and disability data, and avoiding bias introduced when screener questions pre-define respondents [12].

From as early as 2016, WHO member states began requesting a brief version of the MDS to be used as a module that could be integrated into existing surveys [1]. So used, the brief version can estimate and monitor changes in disability prevalence in the general population – when added to health and household surveys – or in subpopulations – when added to specific surveys, such as labour force surveys. It is also useful for monitoring the implementation of international agreements, such as the Sustainable Development Goals (SDGs) and the UN Convention on the Rights of Persons with Disabilities (CRPD). Finally, a brief version of the MDS can also be valuable for collecting information on environmental barriers and facilitators for evidence-informed policymaking, and for estimating rehabilitation needs.

The development of the brief MDS version involved a participatory expert consensus process and analytical work. A technical expert consultation on functioning and disability measurement took place in December 2015 at the WHO Headquarters in Geneva, Switzerland. The WHO Unit on Sensory Functions, Disability and Rehabilitation organized the consultation, in close collaboration with the Surveys, Measurement and Analysis Unit, as well as the Mental Health and Substance Abuse and the Ageing and Life Course Departments. The experts selected questions and reviewed them in terms of their social and cultural universality, overlap with the minimal ICF generic set [13] and statistical criteria. In a second step, the robustness and reliability of the selection the expert proposed were tested using Generalized Partial Credit Model (GPCM) and Bayesian models adjusted for age, gender and income were estimated [14].

The Brief MDS collects information on intrinsic capacity and performance to capture the experience of disability, using selected questions from the original MDS. Intrinsic capacity is defined as all the physical and mental capabilities of an individual and performance as the outcome of the interaction between the individual’s intrinsic capacity and facilitating or hindering features of the physical, human-built, attitudinal and socio-political environment. In addition, the brief MDS also includes questions from the MDS’s sections on health conditions and environmental factors.

Valid metrical information of intrinsic capacity and performance is an essential feature of the MDS. Interval-scaled metrics of intrinsic capacity and performance are constructed and partitioned using ‘fit for purpose’ cut-offs to define severe, moderate and mild levels of difficulties in capacity as well as severe, moderate, and mild levels of disability (using the performance scale), for data disaggregation purposes. It is important therefore that this feature is shared by the brief version as well. It is also important that the Brief MDS measures functioning in a manner that is both simple and easy, but also reliable and valid. This paper has two objectives: 1) to evaluate the metric properties of the capacity and performance metrics generated by the Brief MDS; and 2) to evaluate the precision of the Brief MDS in comparison with the full MDS by quantifying the strength of association between capacity and performance overall scores generated with both versions.

## Methods

The psychometric properties of the brief MDS were evaluated by carrying out a secondary data analyses from two national MDS implementations in 2015: the second national disability survey of Chile (ENDISC II), including a sample of 12,265 adults aged 18 years or older from 15 provinces; and in Sri Lanka, including a sample of 3000 adults aged 18 years or older from all provinces of the country. The data set from Chile is public domain and available at https://www.senadis.gob.cl/pag/355/1197/ii_estudio_nacional_de_discapacidad. The data set from Sri Lanka can be made available by WHO on request, conditional on approval by the country representatives. R code for the analyses are provided in Supplementary material 1.

The brief version of the MDS includes the mandatory MDS modules on environmental factors, performance, capacity and health conditions (the inclusion of questions about health conditions is not necessary if the brief MDS is added to health surveys), but contains a reduced number of questions, and is available online [1].

Table 1 shows the performance and capacity questions included in the brief MDS. The response options range from no problem (0) to extreme problem (4).

**Table 1:** Performance (Module 4000) and capacity (Module 5000) questions included in the metric analyses and included in the final version of the brief MDS

Item number	Questions used in the metric analyses	Questions reworded in the final brief MDS version
**Module 4000 – Functioning (performance)**		
B4001	How much of a problem is walking a kilometer for you?	
B4002	How much of a problem is getting where you want to go for you?	
B4003	How much of a problem is being clean and dressed?	
B4004	How much of a problem is toileting?	
B4005	How much of a problem is looking after your health, eating well, exercising or taking your medicines?	
B4006	How much of a problem is feeling tired and not having enough energy?	
B4007	How much of a problem is coping with all the things you have to do?	
B4008	How much of a problem is remembering to do the important things in your day-to-day life?	
B4009	How much of a problem do you have with getting your household tasks done?	
B4010	How much of a problem do you have with joining community activities, such as festivities, religious or other activities?	
B4011	How much of a problem is getting things done as required at work or school?	
B4012	How much of a problem is using public or private transportation?	
**Module 5000 – Capacity**		
B5002	Do you have difficulty seeing, even if wearing glasses?	How much difficulty do you have seeing things at a distance [without glasses]?
B5003	Do you have difficulty hearing, even if using a hearing aid?	How much difficulty do you have hearing [without hearing aids]?
B5004	Do you have difficulty walking or climbing steps?	
B5005	Do you have difficulty remembering or concentrating?	
B5006	Do you have difficulty (with self-care such as) washing all over or dressing?	How much difficulty do you have washing all over or dressing?
B5007	How much difficulty do you have sleeping because of your health?	
B5008	How much difficulty do you have doing house tasks because of your health?	
B5009	Because of your health, how much difficulty do you have with joining community activities, such as festivities, religious or other activities?	
B5010	How much difficulty do you have with feeling sad, low or depressed because of your health? How much difficulty do you have with feeling worried, nervous or anxious because of your health?	How much difficulty do you have with feeling sad, low, worried or anxious because of your health?
B5011	Because of your health, how much difficulty do you have getting along with people who are close to you, including your family and friends?	
B5012	How much bodily aches or pain do you have?	

### Intrinsic capacity and performance metrics

The Brief MDS, similar to its full counterpart, generates two metrics: one of intrinsic capacity using questions B5002 to B5012 and one of performance using questions B4001 to B4012 (Table 1).

A unidimensional model for polytomous data from the Rasch family, namely the partial credit model (PCM) was used to create the scales and to evaluate their psychometric properties [15]. The PCM was preferred to a Rating Scale Model, as the difficulty thresholds were not observed to be equally distant across items. Further, a PCM was preferred to a generalized (G-)PCM, because the WHO aims, in the long term, to have available capacity and performance metrics that are useful across countries. The expected score sufficiency, i.e. a specific score has only one ability level, is only given by models from the Rasch family that assume equal item discrimination across a scale.

The PCM is based on the assumption that there is a unidimensional latent construct to be measured (in the present case, respectively, intrinsic capacity and performance) and that both person ability and item difficulty estimates can be located along a continuum of this construct. For each person in a sample an interval scaled ability estimate using logits is obtained. This ability estimate represents each person on the continuum of the construct of interest, ranging from low to high disability levels. For each question, the item difficulty estimate gives the location of the question on the same continuum. In addition, with polytomous response options questions’ thresholds are estimated. In the MDS, items are scored on an ordinal rating scale. The functioning scale assesses problems ranging from 1 = none to 5 = Extreme, and the capacity scale assesses difficulties from 1 = none to 5 = extreme. The thresholds are the equal probability point between two response options, so that for a question with k response options, k − 1 thresholds are provided. The ordering of item thresholds is important and should show strictly increasing values, reflecting the intended ordering of the response categories [16]. Monotonicity was tested by observing the ordering of the item difficulty thresholds and category probability curves. In case of disordered thresholds, response options were collapsed as recommended [17]. For example, if the five response options, ranging from 0 to 4, have all the middle categories collapsed because of disordering, the item’s threshold parameterization will be reported as 01112; if only the two most extreme responses are collapsed the item will be reported as coded 01233.

A metric has a good targeting when the estimated difficulty thresholds and the ability estimates tally. Technically, the mean ability and the mean difficulty should both be close to zero. Targeting between item thresholds and persons’ abilities was examined by comparing their distribution along the latent trait continuums. Questions with overlapping thresholds are considered redundant.

Items are expected to be locally independent and not associated above a predefined cut-off. Local independence was examined based on the correlations of the standardized residuals among questions resulting from the PCM analysis [15]. Correlations of the standardized Rasch-residuals of r > 0.2 or higher can be deemed significant with high confidence and indicate redundancy among items [16, 17]. In presence of locally dependent items, those where aggregated into testlets by summing up the respective individual scores [18].

Item fit was examined based on the infit mean square statistics: values between 0.8 and 1.2 indicated good item fit [19]. Infit and Outfit values below 0.8 are not seen as degrading the quality of the Rasch analysis, however it can be expected that the concerned items will be less able to discriminate among middle levels of difficulty because they add little information.

Unidimensionality was tested with bifactor analysis [20–22] and the fulfilment of two conditions: (1) all items load high on a general factor; and (2) factor loadings of questions on the general factor exceed those of group factors. Bifactor analysis was applied on a polychoric correlation matrix – a measure of association for ordinal scaled variables. More details can be found in Supplementary file 2.

In the Rasch model, for a given level of ability the questions should work in a same way irrespective of the group being assessed, i.e. the difficulty of an item should be the same regardless of e.g. age or gender. Items that violate this criterion have Differential Item Functioning (DIF). DIF was tested for age groups (17.5 < years ≤39.5; 39.5 < years ≤59.5; 59.5 < years ≤100) and gender with two methodologies. First, an iterative hybrid ordinal logistic regression was applied to the raw data with a change in McFadden’s pseudo R-squared measure (> 0.02) as DIF criterion [23]. Second, to confirm the findings of the hybrid ordinal logistic regression and get a deeper understanding of some group effects DIF was tested with an ANOVA of the standardized residuals [24]. DIF is reported but item adjustment in presence for DIF for age or gender are not undertaken. The aim of the DIF analysis in this study is to gain insights and to know which items have DIF, to understand if the DIF is mainly due to a true difference in capacity or performance due to age or gender or if DIF indicates that the item is unfairly treating individuals from the different subgroups.

Reliability was evaluated with the Person Separation Index (PSI), which is analogous to Cronbach’s alpha in traditional test theory. Values > 0.7 are considered sufficient for population data, while values > 0.85 are good for individual (clinical) data [24].

The logit scaled ability continuum, where for each respective row score one unique ability parameter is found, was rescaled into a more intuitive scale ranging from 0 (no difficulties in intrinsic capacity; no performance problems) to 100 (extreme difficulties in intrinsic capacity; extreme problems in performance).

Original performance and intrinsic capacity scores of the full MDS version, estimated using PCM and the same procedure described above were carried out by the Chilean Statistics Bureau in collaboration with the WHO for Chile and by WHO headquarters for Sri Lanka. To evaluate the precision of the Brief MDS in comparison with the full MDS, the grade of correspondence between scores generated by the full and brief version was first examined with correlation coefficients. Secondly, to quantify how much of the variability in original performance and intrinsic capacity scores is explained by the brief MDS, we applied linear regression using the original scores as dependent variables and the items of the corresponding brief versions as independent variables. Different collapsing strategies of response options across countries are not a problem because the metrics are created for each country independently. On the same token, cut-offs for mild, moderate and severe disability are defined for each country based on their distribution of performance scores. For instance, the cut-off for severe disability is the sum of the mean and one standard deviation of the performance distribution. What is compared across countries are percentages of persons above certain cut-offs, for instance the percentage of persons with severe disability. The distributions are always anchored at 0 (lowest possible Rasch-based ability), representing no problems on all items and 100, representing the maximum of possible problems (maximum possible Rasch-based ability).

The full and brief MDS versions are available at: https://www.who.int/activities/collection-of-data-on-disability

Data analyses were performed with R [25].

## Results

Table 2 summarizes the demographic characteristics of the samples.

**Table 2:** Sociodemographic characteristics of the samples used in the metric analyses

Sociodemographic Characteristics	Sri Lanka (***N*** = 3000)		Chile (***N*** = 12,265)	
	N	%	N	%
**Gender**				
Male	2602	86,73	5307	43,27
Female	398	13,27	6958	56,73
**Marital Status**				
Never married	395	13,17	3784	30,85
Married	2248	74,93	4324	35,25
Cohabiting	36	1,20	1787	14,57
Separated/divorced	61	2,03	1186	9,67
Widowed	260	8,67	1184	9,65
**Education Level**				
No schooling or no grade completed	116	3,87	1321	10,77
Elementary education	354	11,80	2336	19,05
Vocational education	86	2,87	1201	9,79
Secondary school	2411	80,37	5172	42,17
University	16	0,53	2013	16,41
Post-graduate studies	7	0,23	217	1,77
Other	10	0,33	5	0,04
**Work Situation**				
Working	2699	89,97	7090	57,81

### Intrinsic capacity metric

In the bi-factor analysis of the capacity questions the factor loading on the general factor was consistently higher than the loading on the specific factors, so that the assumption of unidimensionality was met for Chile and Sri Lanka data. In Chile, no local item dependencies were observed, but thresholds were disordered in all items and required recoding of response options (reported in the tables). In Sri Lanka, local item dependency between 5009 *(doing household tasks) and* 5011 (*joining community activities)* and between 5011 (*joining community activities)* and 5015 (*getting along with people who are close)* was observed. Nine items had disordered thresholds and required recoding of response options. Recoding disordered thresholds solved the local dependencies observed at the first iteration. Due to very bad fit and dependency, the items “*Do you have difficulty seeing, even if wearing glasses?”* and “*Do you have difficulty hearing, even if using a hearing aid?”* had to be analysed together as one item with collapsed response options. Final models adjusted for local item dependencies and disordered thresholds showed good infit statistics, below the pre-determined cut-off of 1.2. Outfit statistics above 1.2 indicate a strong influence of outliers with unusual response pattern; this was the case for the item combining the WG questions on seeing and hearing. Table 3 summarizes the results for the capacity metric.

**Table 3:** Results of the polytomous Rasch model for the capacity metric

MDS-Code	Brief MDS Code	Original Questions	Chile						Sri Lanka							
			Recoding strategy	Outfit	Infit	Location	Thresholds		Recoding strategy	Outfit	Infit	Location	Thresholds			
							1	2					1	2	3	4
WG1	B5002	Do you have difficulty seeing, even if wearing glasses?	00122	*1,326*	*1,258*	-1,326	− 2,387	− 0,264	*Collapsed Testlet*	*1,666*	1,119	2,235	2,235			
WG2	B5003	Do you have difficulty hearing, even if using a hearing aid?	00122	1,143	1,153	0,879	0,556	1,203								
WG3	B5004	Do you have difficulty walking or climbing steps?	00122	0,615	0,723	0,060	−0,320	0,441	00122	1,005	1,037	− 0,831	− 1,453	− 0,209		
WG4	B5005	Do you have difficulty remembering or concentrating?	00122	0,870	0,927	0,512	−0,794	1,817	00122	1,011	1,020	0,137	−0,761	1,035		
WG5	B5006	Do you have difficulty (with self-care such as) washing all over or dressing?	00112	0,370	0,691	1,748	1,220	2,277	00122	0,878	0,871	1,559	1,023	2,096		
I5007	B5007	How much difficulty do you have sleeping because of your health?	00122	0,836	0,918	−0,076	−0,662	0,510	00122	1,030	1,079	0,352	−0,239	0,943		
I5009	B5008	How much difficulty do you have doing house tasks because of your health?	00122	0,591	0,713	0,553	0,351	0,754	00122	0,551	0,654	0,448	−0,293	1,190		
I5011	B5009	Because of your health, how much difficulty do you have with joining community activities, such as festivities, religious or other activities?	01112	0,988	0,950	0,471	0,181	0,761	00122	0,641	0,683	0,371	−0,176	0,919		
I5013 & I5014	B5010	How much difficulty do you have with feeling sad, low or depressed because of your health? How much difficulty do you have with feeling worried, nervous or anxious because of your health?	*Collapsed Testlet*	0,894	0,899	−0,640	−2,001	0,720	*Collapsed Testlet*	0,974	1,082	0,108	−0,203	− 1,788	2,467	− 0,044
I5015	B5011	Because of your health, how much difficulty do you have getting along with people who are close to you, including your family and friends?	00122	0,943	1,004	1,547	1,110	1,985	00122	0,610	0,717	0,844	−0,128	1,815		
I5017	B5012	How much bodily aches or pain do you have?	00122	0,846	0,835	− 1,180	− 2,351	− 0,008	00122	1,028	1,037	− 1,135	− 2,413	0,143		

The final model for the Brief MDS capacity scale had sufficient reliability for population studies with a PSI of 0.788 in Chile and 0.838 Sri Lanka, which supports that the measure is sufficiently reliable for population surveys. Explanatory power and strength of the correlations comparing the brief with the full version of the MDS is very good. The brief version explains 98.4% of the variance of the final intrinsic capacity score in the full version in Chile and 92.3% in Sri-Lanka. Correlations between the ability estimates of the two versions were also very good (r = 0.984 Chile; r = 0.838 Sri Lanka) (Table 4).

**Table 4:** Targeting, explanatory power and person separation index (PSI) for the capacity and performance metrics. SD=Standard Deviation

		MDS Chile					MDS Sri Lanka				
		Mean	SD	PSI	Correlation ^a^	Explained variance^b^	Mean	SD	PSI	Correlation ^a^	Explained variance^b^
**Capacity**	**Difficulty**	0,232	1,281	0,788	0,984	0,969	0,293	1,308	0,805	0,838	0,923
	**Ability**	− 1,544	1,338				− 1,189	1,477			
**Performance**	**Difficulty**	0,310	1,258	0,734	0,845	0,715	0,289	1,218	0,805	0,875	0,792
	**Ability**	− 1,623	0,993				− 1,394	1,208			

^a^Correlation Full versus Brief MDS versions’ ability estimates.

^b^Explained variance in predicting the MDS full version ability score based on the brief version’s ability estimates.

### Performance metric

In the bi-factor analysis the factor loading on the general factor was consistently higher than the loading on the specific factors, so that the assumption of unidimensionality was met in both Chile and Sri Lanka. In Chile local item dependency was observed between 4005 (*walking a kilometre) and* 4007 (*getting where you want to go*) (r = 0.220), and between 4010 (*being clean and dressed)* and 4012 (*toileting)* (r = 274). These items were therefore collapsed as testlets in the final model of performance. The original scale was characterized by disordered thresholds in 9 of the items, so that collapsing of response options was necessary. In Sri-Lanka local item dependency was observed between 4005 (*walking a kilometre)* and I4007 (*getting where you want to go)* (r = 0.229). In the first iteration the dependency between 4010 (*being clean and dressed)* and 4012 (*toileting)* was below the pre-defined cut-off (r = 0.108). However, after adjusting the scale in a second iteration the dependency increased above 0.2 so that, as for Chile, these two pair of items entered the final Rasch analysis as testlets. The original scale was characterized by disordered thresholds in all but one item, so that collapsing of response options was necessary. The applied response option collapsing strategies, coded as described in the methods, is found in Tables 3 and 5.

**Table 5:** Results of the polytomous Rasch model for the performance metric

MDS-Code	Brief MDS Code	Question		Recoding strategy	Outfit	Infit	Location	Thresholds							
								1	2	3	4	5	6	7	8
I4005 & I4007	B4001 & B4002	How much of a problem is walking a kilometer for you? How much of a problem is getting where you want to go for you?	**Chile**	012345678	0,724	0,786	−0,479	−0,659	− 1,382	− 0,743	− 0,838	− 0,319	− 0,435	0,433	0,112
			**Sri Lanka**	012345678	0,773	0,797	−0,685	− 1,098	− 1,679	− 1,062	− 0,949	− 0,476	− 0,597	− 0,118	0,501
I4010 & I4012	B4003 & B4004	How much of a problem is being clean and dressed? How much of a problem is toileting?	**Chile**	012345678	0,433	0,645	0,594	1,559	− 0,661	0,528	0,027	1,159	0,304	1,695	0,142
			**Sri Lanka**	012345678	0,973	0,953	0,940	1,395	−0,435	0,610	0,915	1,592	0,828	1,536	1,079
I4014	B4005	How much of a problem is looking after your health, eating well, exercising or taking your medicines?	**Chile**	00122	0,649	0,761	0,527	0,247	0,806						
			**Sri Lanka**	00122	0,563	0,665	1,089	0,504	1,673						
I4021	B4006	How much of a problem is feeling tired and not having enough energy?	**Chile**	01234	0,998	1,026	−0,458	− 1,903	− 0,981	0,100	0,953				
			**Sri Lanka**	00122	0,963	0,952	−0,881	− 1,506	− 0,255						
I4031	B4007	How much of a problem is coping with all the things you have to do?	**Chile**	01112	0,859	0,872	0,588	−0,798	1,974						
			**Sri Lanka**	00122	1,028	0,957	0,312	−0,341	0,965						
I4035	B4008	How much of a problem is remembering to do the important things in your day-to-day life?	**Chile**	01234	1,119	1,163	0,370	−0,461	0,135	0,819	0,988				
			**Sri Lanka**	00122	1,160	1,157	0,286	−0,414	0,987						
I4037	B4009	How much of a problem do you have with getting your household tasks done?	**Chile**	00122	0,680	0,747	0,150	−0,106	0,407						
			**Sri Lanka**	00122	0,616	0,683	0,104	−0,350	0,559						
I4040	B4010	How much of a problem do you have with joining community activities, such as festivities, religious or other activities?	**Chile**	01112	1,179	1,063	0,083	−0,263	0,429						
			**Sri Lanka**	00122	0,784	0,801	0,147	−0,152	0,446						
I4045 & I4047	B4011	How much of a problem is getting things done as required at work/school?	**Chile**	*Collapsed Testlet*	1,290	1,282	2,762	1,186	1,488	5,613					
			**Sri Lanka**	*Collapsed Testlet*	*2,311*	1,371	3,064	1,672	4,456						
I4048	B4012	How much of a problem is using public or private transportation?	**Chile**	01112	0,880	0,859	−0,038	−0,668	0,591						
			**Sri Lanka**	00122	0,986	0,935	−0,522	−0,873	− 0,171						

The performance metric of Chile did not present any misfit. However, for Sri Lanka the item aggregating 4045 (*walking a kilometre)* and 4047 (*getting things done as required at work or school)* showed initially misfit but a borderline acceptable infit of 1.37 could be achieved after adjustment of the metric. In both Chile and Sri Lanka, a few items showed infit and outfit statistics indicating overfit, i.e. a lack of ability in determining middle levels of problems, namely the testlet aggregating 4010 (*being clean and dressed)* and 4012 (*toileting)* as well as 4037 (*getting your house tasks done).* Table 5 summarizes the results for the capacity metric.

The final model for the performance metric has good reliability with PSI being 0.734 in Chile and 0.875 in Sri Lanka, which, as for the capacity metric, supports sufficient reliability for use in population surveys. The high PSI of 0.875 in Sri Lanka would also support its reliable use for measurement and comparisons at individual levels (Table 4). Explanatory power and strength of the correlations comparing the brief with the full version of the MDS is very good. The brief version explains 71.5% of the variance in the full version in Chile and 79.2% in Sri-Lanka. Correlations between the ability estimates of the two versions were also good (r = 0.845 Chile; r = 0.875 Sri Lanka) (Table 4). It is important to stress that the loss in explained variance and correlational strength must be seen in relation with the proportions of items which were removed from the capacity and performance modules of the original MDS. While the Brief MDS capacity metric still includes 72% of the items from the full version, the Brief MDS performance metric includes 30% of the items from the full version.

All DIF analyses showed no DIF by gender in any of the items from the brief capacity and brief performance metrics. DIF by age is somehow expected, given the potentially disabling health losses associated to the ageing process, and do not pose a threat to the questionnaire validity. Nevertheless, it is important to report which items showed age DIF. Some DIF by age groups was found for the item B5002 “Do you have difficulty seeing, even if wearing glasses?” for capacity in Chile and for the testlet grouping B5002 with B5003 “Do you have difficulty hearing, even if using a hearing aids?” in Sri Lanka. DIF by age groups was observed for the performance in Chile and Sri Lanka for the testlet grouping B4001 “How much of a problem is walking a kilometer for you?” and B4002 “How much of a problem is getting where you want to go for you?”, for item B4007 “How much of a problem is coping with all the things you have to do?”, and for the “aggregated” item B4011 “How much of a problem is getting things done as required at work/school?”

## Discussion

The Brief MDS has been developed to provide member states with a short but powerful tool to monitor functioning and disability over time by means of household surveys, and especially health surveys. In this paper, we show that the Brief MDS generates valid metrics for measuring disability, from the perspectives of capacity and performance, thereby achieving good levels of measurement precision in comparison with its full counterpart. Given the scarcity of valid functioning and disability modules for household surveys, the Brief MDS represents a milestone in disability measurement. The Brief MDS can be used by countries who need to monitor disability trends over time, which is especially important to evaluate the impact of health policies and public health interventions on people experiencing different levels of disability, and when disaggregating SDGs and CRPD indicators by disability. By collecting essential information on the needs and barriers facing persons who have specific health conditions, for instance NCDs, the Brief MDS can inform evidence-based policy-making, such as the targeted planning of rehabilitation services. Additionally, the Brief MDS can serve as a starting point for countries interested in developing their own disability modules for household surveys.

WHO operationalizes disability in the MDS as the outcome of an interaction between health conditions and the physical, human-built, attitudinal and socio-political environment in which persons live, and as a continuum ranging from low to very high levels of severity. Consequently, disaggregation made possible by both the full and brief versions of the MDS goes far beyond identifying a dichotomous disability status (disabled versus not disabled) and defines subpopulations experiencing no, mild, moderate, and severe levels of disability. This approach makes direct comparisons between these groups possible, unveiling inequalities and identifying group specific needs both at the individual level, for instance the need of rehabilitation services, and at the level of the environment, for instance the need for accessible toilets in households. Recent studies corroborate the value of this approach. In a pilot study of the MDS in Cambodia, accessible transportation was an important barrier to persons experiencing any degree of disability, while social support, attitudes of others and the availability of regular medication played a key role in the lives of persons with moderate to severe disability [7]. A similar study using MDS data from Cameroon showed that transportation had a very high impact for all persons experiencing disability, while barriers in the toilet of the own dwelling and in the dwelling itself importantly hindered persons with moderate to severe disability [8]. A study using data collected with the MDS in Chile focused on identifying environmental determinants of work performance in employees with anxiety or depression [6]. While barriers in transportation and discrimination mostly affected persons with mild to moderate disability, barriers at the workplace and dwelling, as well as the lack of personal assistance hindered persons with severe disability the most [6]. These exemplary studies show that the approach followed by WHO to collect data on disability has very concrete implications for evidence-informed policy-making, and does not leave, as requested by the SDG, anyone behind.

Importantly, data from the Brief MDS can monitor, at the population level, efforts to scale up rehabilitation in health systems, as requested in the Rehabilitation 2030 Call for Action. The value of the Brief MDS here follows from how WHO defines rehabilitation, namely as “*a set of interventions designed to optimize functioning and reduce disability in individuals with health conditions in interaction with their environment*”. An essential part of the Call for Action is that Health Information Systems are reshaped or expanded in order to accommodate functioning data [26, 27]. However, currently the routine and standardised collection of functioning data in Health Information Systems is rare and, where it exists, usually poorly uncoordinated. Collecting functioning data poses challenges to countries, especially in low resource settings, since it requires collecting information about body functions, activities and participation, and environmental factors (e.g. assistive technology or accessible environments). The Brief MDS collects these data. Information obtained by means of an add-on Brief MDS module incorporated into existing household surveys can therefore considerably improve -- at a fraction of the cost required to implement a stand-alone survey and with a high level of standardization -- rehabilitation services planning at regional and national level.

This study must be understood in the light of its methodological limitations. First, we had to aggregate the five original response categories of the MDS to fit the Rasch model. Disordering of thresholds can have several reasons: erratic response behaviour, item dependencies, poorly defined or too many categories. It can be noted that the response categories in the MDS are only labelled at the extremes and the middle categories are not further specified, allowing for subjective leeway for interpretation. As response categories can always be collapsed, if needed, we see no reason for changing the original number of response options in the survey. Second, although DIF was observed for age groups, we opted for non-adjustment through item split, as an age gradient is anyway expected and corresponds to reality: the older a person the more likely are problems and difficulties in capacity and performance, respectively. Third, the question on why we have used Rasch instead of other Item Response Theory (IRT) models might arise. Rasch was used due to the need of developing a “universal” metric for a universal survey recommended to be implemented worldwide. In that sense, the main focus was not only the individual scores but also the creation of an objective interval scale for measurement, so that other IRT models with additional parameters are not suitable as they lack score sufficiency.

## Conclusion

The brief version of the WHO and World Bank Model Disability Survey (Brief MDS) provides interested stakeholders with a valid brief data collection tool for monitoring levels of functioning and disability over time as an add-on module for household surveys and other data collection platforms. The Brief MDS can assist countries monitor disability trends over time, which is especially important in order to follow up the impact of health policies and public health interventions on people experiencing different levels of disability. The survey also helps with the disaggregation of SDGs and CRPD indicators by disability. By collecting essential information on the needs and barriers facing persons with specific health conditions who experience disability, the Brief MDS can inform evidence-based policy-making. Finally, the Brief MDS can serve as a starting point for countries interested in developing their own disability modules for existing household surveys. Given the scarcity of disability tools fulfilling fundamental metric properties, the Brief MDS represents a milestone in disability measurement.

## Supplementary Information

**Additional file 1.**

**Additional file 2.**

## Data Availability

Data are available from the authors upon request.

## Abbrevations

WHO: World Health Organization
MDS: Model Disability Survey
GPCM: Generalized Partial Credit Model
CRPD: Convention on the rights of persons with disabilities
SDG: Sustainable Development Goals
